# Filial piety and older adult caregiving among Chinese and Chinese-American families in the United States: a concept analysis

**DOI:** 10.1186/s12912-024-01789-0

**Published:** 2024-02-13

**Authors:** Chunhong Xiao, Patricia A. Patrician, Aoyjai P. Montgomery, Youhua Wang, Rita Jablonski, Adelais Markaki

**Affiliations:** 1 University of Alabama at Birmingham School of Nursing, 1720 2nd Avenue South, Birmingham, AL 35294-1210 USA; 2 University of Alabama at Birmingham School of Public Health, 1665, 2nd Avenue South, Birmingham, AL 35294-1210 USA; 3 College of State Governance, Southwest University, No. 2 Tianshen Road, Chongqing, 400715 Beibei District China

**Keywords:** Filial Piety, Chinese and Chinese-American, Older adults, Aging, Caregiving

## Abstract

**Background:**

The culturally sensitive nursing practice has not embedded filial piety as a cultural value and stance pertaining to caregiving among aging Chinese and Chinese-American (CCA) families in the United States, yet it is critical for healthy aging among CCAs.

**Purpose:**

To understand filial piety when caring for aging CCAs and conceptualize an operational definition and framework.

**Methods:**

A systematic search was conducted in CINAHL, PubMed, Scopus, and PsycINFO databases. Analysis of the concept of filial piety among CCAs used Walker and Avant’s methods. Twenty-six studies were selected in the final full-text analysis.

**Findings:**

Synthesis of evidence identified four antecedents: (a) filial obligation as a ‘cultural gene’, (b) sense of altruism, (c) familial solidarity, and (d) societal expectation of ‘birth right’. Attributes included familial material and emotional support, obedience, pious reverence, and societal norms. Consequences were related to caregiver burden, psychological and physical well-being, quality of life, and health equity.

**Conclusion:**

Filial piety is an intrinsic desire to support aging parents and an extrinsic desire to adhere to Chinese societal moral tenets. The proposed operational framework *“Caregiving for aging CCAs in the United States”* merits further study.

## Highlights

• Filial piety attributes are aging-related material and physical support from family and society.

• Filial piety is associated with burden on family caregivers, psychological and physical well-being, quality of life, and health equity.

• Culturally-sensitive healthcare services for aging Chinese and Chinese American individuals and families in the U.S. are hindered by lack of awareness of filial piety's effect.

• Operationalizing filial piety contributes to bridging the gap in nursing knowledge and understanding of the aging needs of Chinese and Chinese Americans in the U.S.

## Introduction

Filial piety is one of the most fundamental, yet relatively unknown, virtues that is universally found in diverse cultures throughout human history [1]. For families of Chinese origin, the concept of filial piety is interwoven into the upbringing and everyday functions of family life [2]. In “The Analects”, Confucius introduces *“Filial piety (a culturally specific term in Chinese, Xiao/Hsiao)”* as *“the source of benevolence and humaneness”* and describes it as an amalgam of physical care, love, service, respect, and obedience. Filial piety requires that adult children care for their aging parents’ physical, emotional and social needs as well as their happiness throughout their lives [3, 4]. The concept embodies three bonding relationships within a family unit: son to father with reverent obedience wife to husband with mutual obligations, and younger to elder with authoritarianism [5]. Inferences from the literature show how filial piety might intersect with other cultures that have similar concepts of caring in the context of family, for example, Asian, African, Latin American, European, Native American, and Muslim cultures [6–9]. Intergenerational responsibility, obligation, reciprocity, and other related concepts are considered filial values [6]. For Chinese and Chinese-American (CCA) families living in the United States (U.S.), filial piety is a psychological factor associated with balancing the traditional Chinese culture with the new host culture [5, 10, 11].

### Dimensions of filial piety

Filial piety is the tenet that has defined familial relationships and caregiving duties across generations of CCA families and includes two dimensions: authoritarian and reciprocal.

*Authoritarian filial piety* is associated with traditional ritual practice. It emphasizes family harmony and parent–child relationships across the lifespan as factors in the modeling of personality [3, 12]. This dimension involves absolute obedience, suppressing individual interests to meet parental needs and happiness, and societal pressure [13–15]. Because authoritarian filial piety requires children to accommodate their parents’ wishes and requirements at the expense of their own desires, it has been identified as a risk factor for CCA caregivers’ mental health [16].

In contrast, *reciprocal* filial piety reflects children’s autogenous gratitude for their parent’s effort in raising them. In return, grown children care for their parents as they age without being restricted by family hierarchy [17]. This investment and reward reflect the attributes and consequences between children and parents, even extended family, such as an uncle, aunt, and siblings. The sense of filial piety stems from grown children’s appraisal of the family environment, thoughts, feelings, and behavior. This is a mutual exchange and accommodation occurring among children and parents in a ‘negotiable’ relationship. Reciprocal filial piety has been shown to be a protective factor for mental health through perceived parental support in muti-social contexts, such as sexual attitudes [18, 19], career adaptation [20], and palliative care options [9, 21].

### Why understanding filial piety is important for nurses?

Given the linguistic and cultural needs, CCAs usually present in healthcare service in dyads; older adults accompanied by family caregivers (usually their adult children). Within aging CCA families, the concept of filial piety is instrumental in determining patterns of caregiving as well as the role of caregivers. Confounded by the belief that only filial persons/children are reliable and trustworthy, it is not uncommon for CCAs to struggle with how to care for an aging family member, as they strive to balance family values with acculturation in the U.S. [16]. Hence, eldercare issues among CCA families are reported to be on the rise [22, 23].

As frontline healthcare personnel, nurses communicate with CCAs and assess their care needs. Understanding filial piety as it pertains to aging and caregiving among CCAs is expected to bridge the cultural chasm in nursing knowledge and improve the quality of rendered services. Accommodating and integrating CCA culture is vital for effectively adapting aging and caregiving practices. Hence, this concept analysis has a two-fold aim: (a) to explore the dimensions of filial piety relevant to caring for aging CCAs as it pertains to nursing, and (b) to propose an operational definition and framework for nurses caring for aging CCA individuals and families.

## Methods

### Concept analysis

Walker and Avant’s [24] eight-step method was chosen to present an overview of applications and define relevant attributes of the concept of filial piety. This deductive method can generate a guide from a general phenomenon to an individual case application [25]. Studies were used to illustrate how filial piety affected the caregiving process, caregiver burden, and caregivers' perceptions towards their elders. Last, an operational framework that described CCA caregiving adaptation was developed based on the most pronounced attributes and consequences.

### Literature search

A literature search was conducted using the following electronic databases: CINAHL PubMed, Scopus, and PsycINFO. In addition, Google Scholar was used to search for literature in the Chinese language. The search terms ‘filial piety’, and ‘Xiao/Hsiao culture’, ‘filial and Chinese aging’, and Chinese words of ‘Xiao’ or ‘Xiaoshun’ were used across all databases in a combination of Boolean strings. Articles written in English or Chinese language with an abstract in English and published from 2001-March 2023 were included. The studies that evaluated life span and care duty from the determinants of intergenerational relationships to guide the care duty were initiated by the book chapter in the Handbook of Chinese Psychology [26] We wanted to keep the literature current as this study took place in March 2023. We also wanted to define antecedents, attributes, and consequences of the concept from studies that more pertain to psychological effects on modern society structure within CCA groups so that the findings were more generalizable and meaningful. Therefore, we started literature published in 2001 after the psychological effect built after Ho’s book was published [26]. SciWheel (a reference manager program) was used for data harmonization, and the suggestion function of SciWheel was used for additional latest publication updates.

**Fig. 1:**
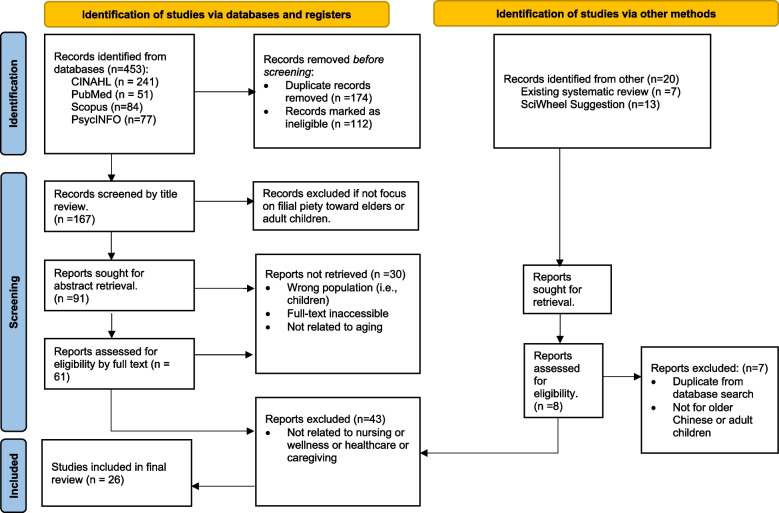
Filial piety among aging Asian families in the United States: Literature search strategy. PRISMA design source [29]

Content inclusion criteria for articles were as follows: (a) focused on Chinese or Chinese-American family, (b) related to a family member living with elders or providing care for elders, (c) related to adult caregivers (over the age of 18 years), and (d) presented a high level of evidence study. Using the adapted *Hierarchy of Evidence for Intervention Studies* classification, the level of evidence was appraised [27, 28]. Articles with a low-level of evidence (level VIII), such as editorials, opinion letters, and expert committee reports, were excluded. The search yielded 258 articles from electronic databases and 20 from other existing systematic review articles and SciWheel suggestions. After removing duplicates and reviewing titles and abstracts, 69 full-text articles remained. A total of 26 studies met inclusion criteria and were chosen for synthesis, as shown in the PRISMA flow chart (see Fig. 1 below).

### Findings

**Fig. 2:**
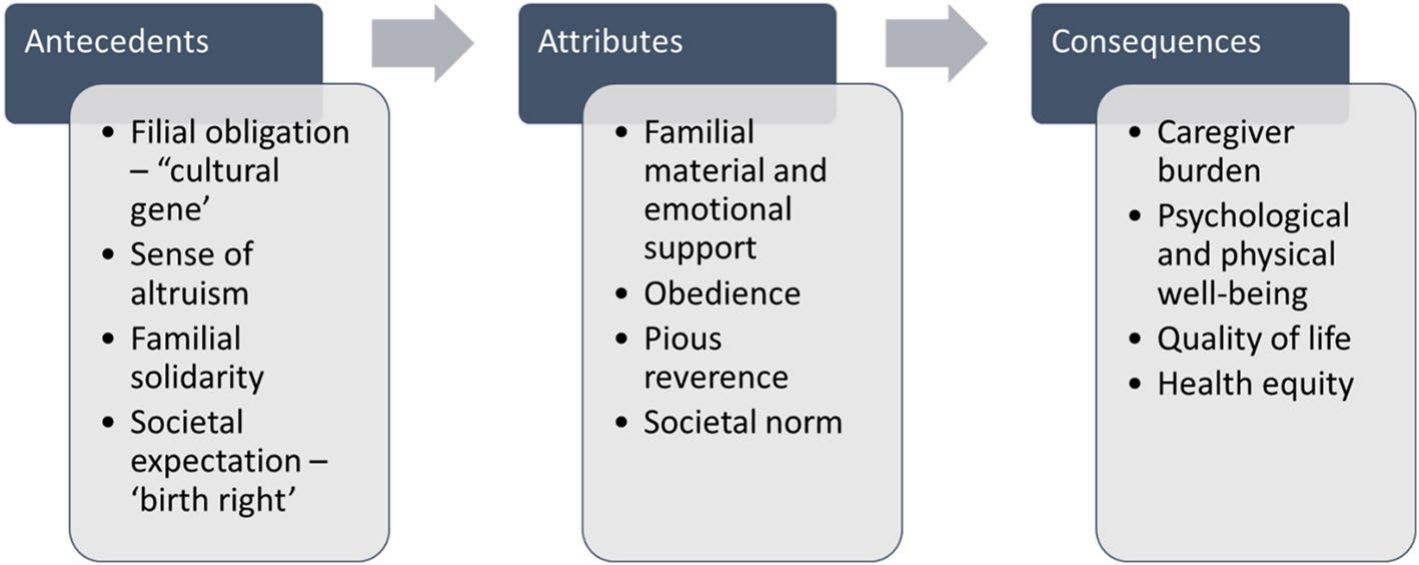
Concept map: Filial piety in caring for aging CCA individuals and families

Synthesis of evidence from the sampled 26 articles identified antecedents (Table 1), defining attributes (Table 2), and consequences (Table 3) for the concept of filial piety in caring for aging CCA individuals and families. The concept map of filial piety in caring for older CCAs is presented in Fig. 2.

**Table 1:** Antecedents of filial piety in caregiving/nursing

Antecedents	Caregiving/nursing	Author&references	Country of origin
Filial obligation- “cultural gene’	Long-term care and nursing home placement, and palliative care	Ho et al., (2012) [30], Feldman&Laland (1996) [31], Gintis (1996) [32]	China USA
Sense of altruism	Culturally appropriate health care services	Zhang et al., (2019) [4], Zhang (2019) [33], Chow (2006) [34]	USA China
Familial solidarity	Elder parent-adult child relations	Guo M., et al. (2019) [11]	USA
Societal expectations- ‘birth right’	Acculturation Indebtedness Family tradition	Stuifbergen (2011) [29] Gui & Koropeckyj-Cox (2016) [30]	Netherlands USA

**Table 2:** Attributes of filial piety to caregiving/nursing among Chinese and Chinese-Americans

Attributes	Caregiving /Nursing	Author &References	Country of origin
Familial material and emotional support	Filial piety medicine; material and emotional support	Bedfird&Yeh (2019) [31, 32] Sringernyuang & Sottiyotin (2022) [33]	Thailand
Obedience	Family relationship assessment	Choy et al., (2018) [34]	USA
Pious reverence	Filial behavior; Modify the ways of providing care	Liu et al. (2011) [13], Luo et al. (2022) [35], Lai et al. (2022) [36], Montayre et al. (2022) [37]	China New Zealand
Societal norm	Caring for the aged	Montayre et al. (2022) [37]	New Zealand

**Table 3:** Consequences of filial discrepancy between caregiver and care recipient among Chinese and Chinese-Americans

Consequences (dyadic well-being)	Well-being components	Author/References	Country of origin
Caregiver burden	Development, emotional, physical, and objective burdens	Guo M., et al. (2019) [16], Lai (2010) [36], Lee et al. (2018) [38], Khalaila (2022) [7], Ng et al. (2016) [39], Yu et al. (2016) [40], Li&Yu (2018) [41], Zhou et al. (2020) [42], Liu&Bern(2020) [43]	USA Israel Singapore
Psychological and physical well-being	Suicidal ideation Depression symptoms Anxiety Stress Loneliness Abusive behavior	Han et al. (2018) [22], Lam et al. (2022) [37], Kim&Silverstein (2020) [44], Liu et al. (2020) [43], Simon, M.A., et al. (2014) [45], Li & Dong (2018) [46], Liu et al. (2019) [47], Liu&Huang (2009) [48], Wang et al. (2014) [49], Xu et al. (2022) [50], Hodgdon&Wong (2019) [51], Zhang et al. (2019) [52]	USA China
Quality of life	Expectations and receipts	Guo et al., (2019) [16], Guo et al., (2020) [53], Liu et al., (2020) [54], Jin et al., (2019) [55]	USA
Health equity	Culturally sensitive community health	Sun et al. (2019) [10], Choy (2018) [56], Wu et al. (2018) [57], Jang et al. (2019) [58], Perreia&Pedroza (2019) [59]	USA

### Antecedents

Based on Walker and Avant [24], antecedents were defined as events that must happen before the occurrence of filial piety. Table 1 represents data extracted from literature that informed antecedents. Initiation of filial piety, as defined by Ho, was found to be triggered by four antecedent conditions [30]. First, thousands of years of Chinese heritage have shaped a ‘cultural gene’ of filial obligation within the family and extended society [30–32]. Second, parental altruism towards children has created harmonious family relationships from generation to generation [4, 33, 34]. Third, familial solidarity formulates individual strength into a higher level of family cohesiveness, especially when facing difficulties [16]. Lastly, societal expectations require that children show affection to elders as a birth right [60, 61]. CCAs have experienced these antecedents before they consider long-term care, nursing home placement, or palliative care. We used definitions and examples in the systematic records (Table 1) to define a cluster of antecedents.

### Defining attributes

Defining attributes encompass the core of a concept and allow for easy identification [24]. Table 2 represents four attributes of filial piety that emerged: familial material and emotional support, obedience, pious reverence, and societal norm [3, 62, 63]. The first attribute described *filial relationship* as an age-based obligation to structure behavior towards caring parents. The second attribute, *obedience,* pertained to the hierarchy between older and younger generations [56]. *Pious reverence* referred to loyalty in family dynamics, requiring devotion and trust for family sustainability [13, 35, 37]. Lastly, filial piety encompassed *societal stability and norms* based on family order, solidarity, and harmony [64]. These attributes are associated with how caregivers respect “filial piety medicine,” assess their family relationships, act with filial duty, and adapt their care provision.

### Consequences

Based on sampled articles, filial piety had four main consequences on CCA adult caregivers: burden, psychological and physical well-being, quality of life, and health equity. Table 3 represents data extracted from literature that informed those consequences.

#### Caregiver burden

Perception of caregiver burden was a sense of filial obligation towards meeting tangible and intangible needs in care, decision-making, and emotional support [7, 16, 36, 38–41]; we found that filial piety mediated or partially mediated the caregiving burden and gain among caregivers. The burden of practicing filial piety was moderated by the sense of honor and reward [42]. A strong sense of filial obligation was significantly and positively associated with lower levels of developmental and emotional burden [16, 39]. However, some caregiving burdens were unavoidable, such as physical burden or the pressure of time when there were personal needs to be met. This objective burden presented a dilemma for families with only one child and families living in different locations [16]. Even though caregiver perception of the burden or caregiving experience varied, children who did not honor filial piety displayed an increasingly strong sense of guilt [43]. Several CCAs realized that they needed to adjust their expectations and understanding of practicing filial piety by simplifying parents’ and adult children’s physical and mental needs.

#### Psychological and physical well-being

Filial piety caregivers reported a feeling of coercion, depression, anxiety, stress, abusive behavior, and even suicidal ideation stemming from their family, society, and cultural heritage [37, 44–48, 54]. Cohabitation with older CCAs and institutionalization presented a strain that reduced caregivers’ well-being [49]. Though there was no evidence of association between physical health of CCA caregiver and filial piety, adult CCA caregivers were prone to high blood pressure, diabetes, and heart disease [50, 51]. Adult children who embraced filial piety and tried to meet their parents’ needs, known as Xiaozi, experienced role strain [23, 52]. Depression, anxiety, loneliness, negative or positive emotion, self-esteem and integrity, and sense of happiness were key components of psychological well-being for caregivers practicing filial piety.

#### Quality of life

Findings varied with five articles showing positive effects on caregivers’ quality of life from practicing filial piety [10, 53, 55, 65, 66]. Decision-making regarding life changes was discussed within the family and was shown to benefit family-centered health care [53, 65]. However, financial support and parent-children shared decision-making were limited to some families. Caregivers with limited financial resources reported that following a traditional filial piety structure reduced their quality of life [10]. Housework-help from children showed a more profound impact in quality of life for female caregivers, while financial support provided a better quality of life for male CCAs [66]. Negative effects in family relationships included reduced quality of life, family support and solidarity [55]. Filial behavior and discrepancy between quality of life and received types of support presented a paradox between male and female older CCAs [66].

#### Health equity

Differences between aging in place (co-residing with their adult children) or institutional placement among groups of Chinese descent and their family caregiver dyads were attributed to the influence of filial piety [34, 57, 67]. Use of formal versus informal health care was associated with accessibility to services and resource availability for a CCA family [58]. Several social determinants affected health equity [59]. For example, urban versus rural, well-established versus developing CCA communities, and second-generation U.S.-based CCAs versus first-generation CCAs who had originally immigrated to the U.S. [13, 58, 68].

### Exemplars

The story of Andrew, presented as an exemplar in Table 4 and developed according to Walker & Avant’s methods, illustrates all the defining attributes of the concept of filial piety. This model case illustrates filial piety as the pillar of family structure. Andrew showed respect, kindness, and dedication to his parents by providing material and emotional support. In respect of his father’s superior position as an elder, Andrew included him in decision-making, trusting his father’s rational advice and wisdom. A parent’s words are orders, and the level of being filial is measured by following the parent’s words. Taking care of his father at home demonstrated societal mores and home stability. Andrew’s case provides a lens of viewing an authoritative filial relationship between a CCA parent and adult child.

**Table 4:** Model case [69]

Andrew, a 30-year-old man from Hong Kong with a family of his own, became a caregiver for his 64-year-old father. Andrew believed it was his duty to care for his father. Andrew discussed with his father about taking a leave of absence from work, so he could stay home and become his father’s caregiver. Together they worked out a plan and the father was very pleased and grateful. Andrew believed that the family grew stronger by practicing filial piety. Andrew considered filial piety not as a choice but rather as a moral obligation	

Alternatively, the borderline case story of Lee, presented in Table 5, contains most of the defining attributes, but not all of them. Intrinsically, Lee is motivated to care for her father as a family member and feels a sense of filial piety. Extrinsically, Lee is motivated to visit her father to prevent feeling social pressure and being criticized as an unfilial child. Lee’s case also reflects the reciprocal dimension of filial piety when a CCA parent and adult child prefer to jointly assess their needs and communication options to accommodate reality, respectively.

**Table 5:** Borderline case [69]

Lee, a 50-year-old daughter, chose to institutionalize her 81-year-old father for supportive care. She respects her father and has great gratitude, but her filial piety is not absolute. Lee believed that living with her father and physically taking care of him was not an obligation. Lee viewed her father as an individual with different needs. Therefore, Lee decided to use a nursing home facility for her father’s long term care needs. Lee visits her father every day, no matter how busy she is. She says that making time to see him is all she can do	

In contrast, the case story of Mr. A, presented in Table 6, contains none of the defining attributes, showing the outcome in the absence of filial piety. In the aging process, Mr. A's parents held onto their expectations rooted in the sense of filial piety in the family. Older CCAs and their adult children's unmatched knowledge and perception of filial relations brought a sense of care responsibility with full care needs and resources (material and psychological).

**Table 6:** Contrary case [Hypothetical case]

Mr. A was adopted by a CCA family and grew up in the U.S. Mr. A did not believe in filial piety and lived in a different house from his adopted parents. Mr. A visited his dad/ mom twice a year. When Mr. A was asked to make decisions about caregiving for his parents, who were diagnosed with Alzheimer’s, he stated that his parents should receive institutional care with payment from their savings. Mr. A believed that the primary consideration should be for people to live the way they want. He did not understand filial piety, nor feel any duty towards his parents. Ultimately, the parents committed suicide, leaving a will where they expressed their disappointment with their adopted son. The CCA parents despaired for their quality of life in the near future due to the Alzheimer’s progression	

In summary, the model and borderline cases exemplify different degrees of and incentives for filial piety. Andrew desired to live with his father and personally take care of him physically. His commitment to filial piety is seen as a moral imperative. He knew that the honor of caring for his father would outweigh any burden he might experience. In contrast, Lee’s view of filial piety as not a moral imperative allows her to verbalize a rationale for having her father cared for by others. Last, the hypothetical case is based on previous studies which have demonstrated a high risk of suicide ideation among older CCA recipients in the United States [45, 70].

## Discussion

This concept analysis explored filial piety through the lens of caregiving for aging CCAs in the U.S. Our aim was to explore the dimensions of filial piety relevant to caring for aging CCAs, with the intent of proposing an operational definition and framework for nurses. Filial piety was defined as an ethical and cultural component that influences CCA relationships and authority within a family and beyond [26, 34, 71]. The attributes of intergenerational filial relationships were found to be the guiding principles governing the Chinese pattern of socialization throughout the lifespan [72]. In the context of a multicultural society in the U.S., acculturation was viewed both as a strength and a weakness which might conflict with a family’s linear bond to filial piety [73, 74]. The moral and social function of filial piety was often ignored as common sense or a “ritual” among CCAs and Asian society.

The antecedents identified in our review are not limited to the CCA community but are also demonstrated in other cultures. For instance, researchers found that authoritarian filial piety is different across cultures and reflects the birthright and indebtedness of CCA children to their older parents, especially those who were born and grew up in mainland China [75]. Li and colleagues demonstrated that Chinese culture influences intergenerational responsibilities through filial piety across Asian countries such as Singapore, Thailand, and Malaysia [9]. Similarly, Khalaila highlighted the positive effect of dual filial piety and how the underlying mechanisms worked against the harmful effects of caregiving stressors in traditional societies [7]. Richardson found strong negative feelings about nursing homes in Korean and Hispanic cultural-based groups, while also feeling isolated and without alternatives in caring for older adults [76]. In Western cultures, most CCAs are either Christian or non-religious, and being pious to the family is critical to caregiving attitudes and behavior. The nature of child-parent relationships implied by filial piety is particularly important in aging care activities from children to their parents [6]. Whereas, in countries where Chinese culture is not dominant, CCAs face a dilemma when deciding whether to receive aging care in an institution, at home or even sending aging parents back to their country of origin. Therefore, filial piety is located at the intersection of culture, religion, and spirituality among different ethnic immigrant groups.

This literature review demonstrated a knowledge gap in assessing and appraising family health in terms of caregiving for CCA parents. Although results from the PINE study on CCA aging [77] have generated interest pertaining to CCA aging at the public health level, filial piety has not been examined as an important scientific concept in nursing care. The psychological influence of filial piety has been demonstrated by its moderating effect on culture in a palliative study [9]. Results from community-based participatory research suggested that being a receiver of filial piety was a risk factor for suicidal ideation [45] and negatively affected the elder CCAs’ life satisfaction [10]. Because of a strong sense of filial obligation [78], CCAs were most likely to care for family elders in their homes [33, 79]. Meanwhile, CCAs had a higher risk for elder abuse, negligence, and suicidal ideation [48, 57]. Elder mistreatment/abuse [80, 81] and domestic violence were also related to the perceived cohesive family relationships associated with filial piety, even if that perception of cohesion was emotionally detrimental to caregivers [77, 80]. Compared to other ethnic groups, CCA families were the least likely to use long-term care facilities since the stigma of being an unfilially adult child impacted decision-making for nursing home use [82]. It is notable that in China, parents can initiate a lawsuit if they believe their adult children are not practicing filial piety (Law of LaoNian QuanYi Bao Zhang #14). Yet, caregiving responsibilities for aging parents of CCAs living outside of China are perceived differently.

Among CCA families in the US, filial piety was a fundamental familial lineage and a moral obligation demonstrated by the action of taking care of aging parents. An intergenerational study of immigrant CCAs showed filial piety as a motivating factor for family health and healthy aging [17]. Prioritizing quantity and incorporating realistic expectations in cultures that are not dominated by Chinese culture has been suggested as an effective coping strategy for promoting older adults’ well-being [83]. Adaptation and appraisal of the care obligation among CCAs in the U.S. were related to older CCAs’ and their adult children’s positive and negative health outcomes. Hence, an operational definition and framework to understanding and embedding filial piety into culturally congruent care for CCA families is proposed below.

### Operational definition and proposed framework

**Fig. 3:**
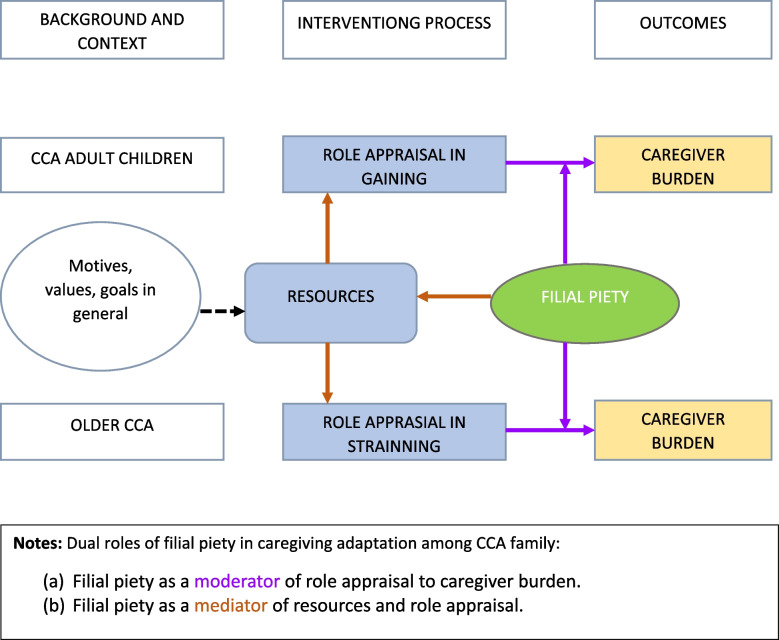
Caregiving for aging CCA individuals and families in the U.S.: An operational framework. Adapted from Kramer [84]

Based on attributes from the synthesis of sampled literature, the following operational Given that knowledge of filial piety is necessary for healthcare providers to address the adaptation of caregiving among CCAs, the investigators proposed an operational definition (Table 7) and framework (Fig. 3) to explain and measure CCA caregiver outcomes. A representation of the proposed framework, entitled *“Caregiving for aging CCA individuals and families in the U.S.”* is shown in Fig. 3. The theoretical underpinning is Kramer’s [84] Conceptual Model of Caregiver Adaptation for understanding the mediation effect of filial piety in caregiving for CCAs. This decision was supported by evidence that people tend to maintain their basic cultural values and behavior regardless of the social framework [85–87]. Therefore, the proposed framework intends to operationalize filial piety as dual roles, a mediator of resources and role appraisal and a moderator of caregiving burden and role appraisal.

**Table 7:** Operational Definition of Filial Piety

Filial piety is an intrinsic desire of adult children to support parents/elders materially and emotionally as well as an extrinsic desire to adhere to the Chinese societal moral tenet to honor family and be honorable children

### Measuring the concept of filial piety

To examine the effect of filial piety in caregiving adaptation, three instruments for measuring filial piety are presented in Table 8.

**Table 8:** Instruments measuring filial piety for CCA caregiving adaptation

Concept	Measuring Instruments
Filial piety	1. *Dual Filial Piety Scale* with two subscales: reciprocal and authoritarian [14] • Scale contains 9-items that overlap between reciprocal filial piety (RFP) and authoritarian filial piety (AFP). For each subscale, there are two sets of 7-itemsthat measure RFP and AFP, respectively. RFP example: “exchange ideas to make decisions.” AFP example: “try my best to complete parents’ unachieved goals.” 2. 10-item *Contemporary Filial Piety Scale (CFPS)* measures filial piety in the twenty-first century (Lum et al., 2016). An exemplar item: “Pragmatic obligation. Arrange care for parents when they can no longer care for themselves.” 3. 19-item *Filial Piety Representations at Parents’ End of Life Scale (FPR-RoL)* measures filial piety expressions of the adult children of Macao Chinese (Che et al., 2022). An exemplar item: “I would choose to temporarily leave my job and concentrate on accompanying and caring for my parents.”

### Strengths and limitations

A primary strength of this work was the analysis of attributes and antecedents of filial piety from the traditional Chinese social context, which provides a reference content of filial piety. The first author, CX, was able to access and synthesize literature in native Chinese language. Limitations include the small number of studies from the caregiving recipient's perspective, which presented a unilateral perception of filial piety. This was not surprising given the scarcity of research on CCAs’ well-being in the U.S. and across healthcare-related research. Second, in searching the literature, potential information bias could have occurred because measures for filial piety do not include contextual factors related to acculturation, growth environment, and societal components [88, 89]. Third, instruments measuring filial piety were old, and not specifically applicable in the U.S. Existing studies have yielded mixed results regarding the effect of filial piety in caregiving outcomes [54, 66, 90, 91]. Finally, given that Kramer’s caregiver adaptation conceptual model has not been tested, the proposed operational framework needs more evidence to support the hypothesis of benefits in the appraisal of caregiver outcomes.

### Implications for future research / recommendations

In spite of the rapid increase of CCAs in the U.S., little is known about filial piety by nurses and other healthcare providers. The majority of U.S. studies were conducted in large cities on the West Coast (i.e., San Francisco), and greater Chicago area where CCAs and other Asian families have access to more caregiving resources. On the contrary, in the Southeastern U.S., there are limited resources to support CCA family caregivers. This lack of knowledge has curtailed introducing this major cultural tenet as part of the standard health assessment of CCA families.

Hence, there is a growing need for providing culturally sensitive health care for the Asian-American population who share the same cultural values. The role of filial piety in caregiver burden remains ambiguous, and the mental health of the CCA family caregiver is unknown. To fill the gaps, future studies should focus on the effect of filial piety in health care literacy and its influence on the well-being of CCA family caregivers. Developing psychometrically sound measurements with contextual factors is critical. Healthcare practitioners in primary care, inpatient, and long-term care settings need training on cultural sensitivity and humility to provide optimal care for older CCAs and their family caregivers. The development of family-centered care interventions is vital for CCA and Asian immigrant families who are most likely living in an intergenerational household.

Nurses, as the most trusted first-line healthcare providers, need to be aware of the importance of filial piety when discussing clinical concerns, care plans, and delegation of care with CCA families. With a rapidly aging population and growing migration trends, there is an undisputed need for raising health professionals’, and particularly nurses’, cultural awareness, competence, sensitivity, and humility in caring for aging immigrant individuals and populations [92, 93]. The International Council of Nurses (ICN), the World Health Organization [WHO], the National Institute of Health (NIH), and the American Association of Colleges of Nursing (AACN) have all called for culturally congruent nursing care to address health care coverage and access inequities [AACN, 2008; ICN, 2007a; Douglas et al., 2014; NIH, 2021; WHO, 2008]. From a nursing perspective, familiarity with the concept of filial piety provides a foundation to offer culturally sensitive services to CCA individuals and their families.

## Conclusion

The permeating cultural value of filial piety affects help-seeking behavior, caregiving, family relationships, and caregiver health for CCA families. The evolution of filial piety has shown significant changes in expectations, attitudes, and emotions toward age-based obligations within immigrant Asian/CCA families undergoing acculturation. Attributes, antecedents, and consequences of filial piety were identified. Both model and borderline exemplars demonstrated the different dimensions of filial piety. Three instruments were found that directly and indirectly measure attributes of filial piety. The proposed operational definition of filial piety and model intends to address the need for culturally sensitive care and support to CCA families and caregivers.

## Data Availability

The datasets used and analyzed during this study are available from the corresponding author.

## Abbreviations

CCA: Chinese and Chinese American
U.S.: United States
ICN: The International Council of Nurses
WHO: The World Health Organization
NIH: The National Institute of Health
AACN: The American Association of Colleges of Nursing

## References

[1] Bedford O, Yeh K-H, Tan C-S (2022). Editorial: filial piety as a universal construct: from cultural norms to psychological motivations. Front Psychol.

[2] Canda ER (2013). Filial piety and care for elders: a contested Confucian virtue reexamined. J Ethn Cult Divers Soc Work.

[3] Bedford O, Yeh K-H (2019). The history and the future of the psychology of filial piety: Chinese norms to contextualized personality construct. Front Psychol.

[4] Zhang M, Lin T, Wang D, Jiao W (2019). Filial piety dilemma solutions in Chinese adult children: The role of contextual theme, filial piety beliefs, and generation. Asian J Soc Psychol..

[5] Ebrey P, Walthall A, Palais J (2009). Confucius and the analects: to 1800: a culture social, and political history. Pre-Modern East Asia.

[6] Jones PS, Lee JW, Zhang XE (2011). Clarifying and measuring filial concepts across five cultural groups. Res Nurs Health.

[7] Khalaila R (2022). The relationship between dual filial piety and caregiver burden among Arab family caregivers in Israel. Res Gerontol Nurs.

[8] Zong X, Ingoglia S, Lo Coco A, Tan J-P, Inguglia C, Liga F (2023). Evaluating the filial behaviour scale across three cultural groups using exploratory structural equation modelling. Int J Psychol.

[9] Li WW, Singh S, Keerthigha C (2021). A cross-cultural study of filial piety and palliative care knowledge: moderating effect of culture and universality of filial piety. Front Psychol.

[10] Sun P, Fan X, Sun Y, Jiang H, Wang L (2019). Relations between dual filial piety and life satisfaction: the mediating roles of individuating autonomy and relating autonomy. Front Psychol.

[11] Wang D, Laidlaw K, Power MJ, Shen J (2009). Older people’s belief of filial piety in China: expectation and non-expectation. Clin Gerontol.

[12] McCrae RR, Costa PT (1997). Personality trait structure as a human universal. Am Psychol.

[13] Liu B-S, Li C-Y, Yeh K-H, Huang H-C (2011). Differences in filial behavior in multigeneration families that live together. J Nurs Res.

[14] Yeh K-H, Bedford O (2003). A test of the dual filial piety model. Asian J Soc Psychol.

[15] Yeh K-H, Yi C-C, Tsao W-C, Wan P-S (2013). Filial piety in contemporary Chinese societies: a comparative study of Taiwan, Hong Kong, and China. Int Sociol.

[16] Guo M, Kim S, Dong X (2019). Sense of filial obligation and caregiving burdens among Chinese immigrants in the United States. J Am Geriatr Soc.

[17] Bedford O, Yeh K-H (2021). Evolution of the conceptualization of filial piety in the global context: from skin to skeleton. Front Psychol.

[18] Liu F, Chui H, Chung MC (2022). Reciprocal/authoritarian filial piety and mental well-being in the Chinese LGB population: the roles of LGB-specific and general interpersonal factors. Arch Sex Behav.

[19] Liu X, Bai S (2022). Mediating effect of filial piety between the elderly’s family resource contribution and their family power: evidence from China. Front Psychol.

[20] Hui T, Yuen M, Chen G (2018). Career-related filial piety and career adaptability in Hong Kong university students. Career Dev Q.

[21] Nation Howard BJ, Shannon RP (2013). Filial piety in palliative care: Faithfully following family feelings. Or is it?. J Palliat Med..

[22] Han M, Diwan S, Sun K (2019). Exploring caregiving-related experiences among Chinese American and European American family caregivers of persons with mental illness. Transcult Psychiatry.

[23] He L, van Heugten K (2020). Chinese migrant workers’ care experiences: a model of the mediating roles of filial piety. Qual Health Res.

[24] Walker LO, Avant KC. Concept analysis. Strategies for theory construction in nursing. 6th ed. Pearson; 2019. p. 157–78.

[25] Azungah T (2018). Qualitative research: deductive and inductive approaches to data analysis. Qual Res J.

[26] Ho DYF, Bond MH (1996). Filial piety and its psychological consequences. The handbook of Chinese psychology.

[27] Dang D, Dearholt SL. Johns Hopkins nursing evidence-based practice: Model and guidelines. Sigma Theta Tau Int. 3rd ed. 2017. ISBN13: 9781940446974.

[28] Fineout-Overholt E, Melnyk BM, Stillwell SB, Williamson KM (2010). Evidence-based practice, step by step: critical appraisal of the evidence: part II: digging deeper–examining the “keeper” studies. Am J Nurs.

[29] Page M, McKenzie J, Bossuyt P, Boutron I, Hoffmann T, Mulrow CD (2021). The PRISMA 2020 statement: an updated guideline for reporting systematic reviews. BMJ.

[30] Ho DYF, Xie W, Liang X, Zeng L (2012). Filial piety and traditional Chinese values: a study of high and mass cultures. Psych J.

[31] Feldman MW, Laland KN (1996). Gene-culture coevolutionary theory. Trends Ecol Evol.

[32] Gintis H (2011). Gene-culture coevolution and the nature of human sociality. Philos Trans R Soc Lond B Biol Sci.

[33] Zhang H (2019). Sending parents to nursing homes is unfilial? An exploratory study on institutional elder care in China. Int Soc Work.

[34] Chow NWS (2006). The practice of filial piety and its impact on long-term care policies for elderly people in Asian Chinese communities. Asian J Gerontol Geriatr.

[35] Luo Y, Wu X, Liao L, Zou H, Zhang L (2022). Children’s filial piety changes life satisfaction of the left-behind elderly in rural areas in China?. Int J Environ Res Public Health..

[36] Lai DWL (2010). Filial piety, caregiving appraisal, and caregiving burden. Res Aging.

[37] Lam JSH, Links PS, Eynan R, Shera W, Tsang AKT, Law S (2022). “I thought that I had to be alive to repay my parents”: filial piety as a risk and protective factor for suicidal behavior in a qualitative study of Chinese women. Transcult Psychiatry.

[38] Lee J, Sohn BK, Lee H, Seong SJ, Park S, Lee J-Y (2018). Attachment style and filial obligation in the burden of caregivers of dementia patients. Arch Gerontol Geriatr.

[39] Ng HY, Griva K, Lim HA, Tan JYS, Mahendran R (2016). The burden of filial piety: a qualitative study on caregiving motivations amongst family caregivers of patients with cancer in Singapore. Psychol Health.

[40] Yu H, Wu L, Chen S, Wu Q, Yang Y, Edwards H (2016). Caregiving burden and gain among adult-child caregivers caring for parents with dementia in China: the partial mediating role of reciprocal filial piety. Int Psychogeriatr.

[41] Li EC-Y, Yu CK-C (2018). Filial piety as a protective factor against burden experienced by family caregivers of diabetic patients in Hong Kong. Asia Pac J Couns Psychother..

[42] Zhou J, Guo Q, Xu R (2020). Reciprocal filial piety facilitates academic success via autonomy: generalizing findings in Chinese society to a global context. Front Psychol.

[43] Liu J, Bern-Klug M (2016). “I should be doing more for my parent:” Chinese adult children’s worry about performance in providing care for their oldest-old parents. Int Psychogeriatr.

[44] Kim JH, Silverstein M (2020). Are filial piety and ethnic community engagement associated with psychological wellbeing among older Chinese American immigrants?. A cultural resource perspective. Res Aging..

[45] Simon MA, Chen R, Chang E-S, Dong X (2014). The association between filial piety and suicidal ideation: findings from a community-dwelling Chinese aging population. J Gerontol A Biol Sci Med Sci.

[46] Li M, Dong X (2018). The association between filial piety and depressive symptoms among U.S. Chinese older adults. Gerontol Geriatr Med..

[47] Liu J, Mao W, Guo M, Xu L, Chi I, Dong X (2019). Loss of friends and psychological well-being of older Chinese immigrants. Aging Ment Health..

[48] Liu BS, Huang HC (2009). Family care for the elderly and the importance of filial piety. Hu Li Za Zhi.

[49] Wang J, Chen T, Han B (2014). Does co-residence with adult children associate with better psychological well-being among the oldest old in China?. Aging Ment Health.

[50] Xu H, Liu J, Zhang Z, Li L (2022). Sandwiched grandparents and biological health risks in China. J Health Soc Behav.

[51] Hodgdon BT, Wong JD (2019). Influences of work and family spillover on filial and sandwiched caregivers’ psychological well-being. Int J Aging Hum Dev..

[52] Zhang W, Liu S, Sun F, Dong X (2019). Neighborhood social cohesion and cognitive function in U.S. Chinese older adults-findings from the PINE study. Aging Ment Health..

[53] Guo M, Byram E, Dong X (2020). Filial expectation among Chinese immigrants in the United States of America: a cohort comparison. Ageing Soc.

[54] Liu J, Wu B, Dong X (2020). Psychological well-being of Chinese immigrant adult-child caregivers: how do filial expectation, self-rated filial performance, and filial discrepancy matter?. Aging Ment Health.

[55] Jin CC, Zhao BB, Zou H (2019). Chinese delinquent and non-delinquent juveniles: an exploration of the relations among interparental intimacy, interparental conflict, filial piety and interpersonal adjustment. Child Youth Serv Rev.

[56] Choy MM. Obeying an evolving cultural value: Influences of filial piety and acculturation on Asian - Americans [Master thesis]. Duke University; 2018.

[57] Wu MH, Chang SM, Chou FH (2018). Systematic literature review and meta-analysis of filial piety and depression in older people. J Transcult Nurs.

[58] Jang Y, Rhee M-K, Cho YJ, Kim MT (2019). Willingness to use a nursing home in Asian Americans. J Immigr Minor Health.

[59] Perreira KM, Pedroza JM (2019). Policies of exclusion: implications for the health of immigrants and their children. Annu Rev Public Health.

[60] Stuifbergen MC, Van Delden JJM (2011). Filial obligations to elderly parents: a duty to care?. Med Health Care Philos.

[61] Gui T, Koropeckyj-Cox T (2016). “I am the only child of my parents:” perspectives on future elder care for parents among Chinese only-children living overseas. J Cross Cult Gerontol.

[62] Lai DWL, Lee VWP, Li J, Dong X (2019). The impact of intergenerational relationship on health and well-being of older Chinese Americans. J Am Geriatr Soc.

[63] Sringernyuang L, Sottiyotin T (2022). “ya luk ka tan yoo”: An ethnography of filial piety culture, medication usage, and health perceptions of the elderly in rural southern Thailand. Int J Environ Res Public Health..

[64] Montayre J, Saravanakumar P, Zhao I, Holroyd E, Adams J, Neville S (2022). Holding on and letting go: views about filial piety among adult children living in New Zealand. J Clin Nurs.

[65] Wang K, Zhang A, Sun F, Hu RX (2020). Self-rated health among older Chinese Americans: The roles of acculturation and family cohesion. J Appl Gerontol..

[66] Ang S, Malhotra R (2022). The filial piety paradox: receiving social support from children can be negatively associated with quality of life. Soc Sci Med.

[67] Chen X, Su D, Chen X, Chen Y (2022). Effect of informal care on health care utilization for the elderly in urban and rural China: evidence from China health and retirement longitudinal study (CHARLS). BMC Health Serv Res.

[68] Lai DWL (2008). Intention of use of long-term care facilities and home support services by Chinese-Canadian family caregivers. Soc Work Health Care.

[69] Chan CLW, Ho AHY, Leung PPY, Chochinov HM, Neimeyer RA, Pang SMC (2012). The blessings and the curses of filial piety on dignity at the end of life: lived experience of Hong Kong Chinese adult children caregivers. J Ethnic Cult Divers Soc Work.

[70] Dong X, Chen R, Wong E, Simon MA (2014). Suicidal ideation in an older U.S. Chinese population. J Aging Health..

[71] Liu F, Ren Z, Chong ESK (2023). On the link between reciprocal/authoritarian filial piety and internalized homonegativity: perceived pressure to get married in a heterosexual marriage as a mediator. Arch Sex Behav.

[72] Qi X (2015). Filial obligation in contemporary China: evolution of the culture-system. J Theory Soc Behav.

[73] Ho GWK (2014). Acculturation and its implications on parenting for Chinese immigrants: a systematic review. J Transcult Nurs.

[74] Chen W-W, Yan JJ, Chen C-C (2018). Lesson of emotions in the family: the role of emotional intelligence in the relation between filial piety and life satisfaction among Taiwanese college students. Asian J Soc Psychol.

[75] Lim AJ, Lau CYH, Cheng C-Y (2021). Applying the dual filial piety model in the United States: a comparison of filial piety between Asian Americans and Caucasian Americans. Front Psychol.

[76] Richardson VE, Fields N, Won S, Bradley E, Gibson A, Rivera G (2019). At the intersection of culture: ethnically diverse dementia caregivers’ service use. Dementia.

[77] Dong X (2014). Do the definitions of elder mistreatment subtypes matter? findings from the PINE study. J Gerontol A Biol Sci Med Sci.

[78] Zhang L, Han Y, Ma Y, Xu Z, Fang Y (2020). Eastern perspectives on roles, responsibilities and filial piety: a case study. Nurs Ethics..

[79] Sun F, Ong R, Burnette D (2012). The influence of ethnicity and culture on dementia caregiving: a review of empirical studies on Chinese Americans. Am J Alzheimers Dis Other Demen.

[80] Dong X, Chang E-S, Wong E, Wong B, Simon MA (2011). How do U.S. Chinese older adults view elder mistreatment? Findings from a community-based participatory research study. J Aging Health..

[81] Li M, Kong D, Chao Y-Y, Dong X (2020). Association between personality traits and elder abuse in a community-dwelling Chinese population: findings from the PINE study. J Elder Abuse Negl..

[82] Luo M, Xue Y, Zhang S, Dong Y, Mo D, Dong W (2018). What factors influence older people’s intention to enroll in nursing homes? A cross-sectional observational study in Shanghai, China. BMJ Open.

[83] Ren P, Emiliussen J, Christiansen R, Engelsen S, Klausen SH (2022). Filial piety, generativity and older adults’ wellbeing and loneliness in Denmark and China. Appl Res Qual Life.

[84] Kramer BJ (1997). Gain in the caregiving experience: where are we? what next?. Gerontologist.

[85] Knight BG, Sayegh P (2010). Cultural values and caregiving: the updated sociocultural stress and coping model. J Gerontol B Psychol Sci Soc Sci.

[86] Mao W, Jia L, Ling X, Chi I (2020). Acculturation and health behaviors among older Chinese immigrants in the United States: A qualitative descriptive study. Nurs Health Sci..

[87] Shafer WE, Fukukawa K, Lee GM (2007). Values and the perceived importance of ethics and social responsibility: The U.S. versus China. J Bus Ethics..

[88] Fu X, Wang F, Chen X, Wei X (2016). Filial piety: theories, measurements, variations, and relationships to related variables. Adv Psychol Sci.

[89] Fu YY, Xu Y, Chui EWT (2020). Development and validation of a filial piety scale for Chinese elders in contemporary China. Int J Aging Hum Dev.

[90] Guo M, Wang L, Day J, Chen Y (2021). The relations of parental autonomy support, parental control, and filial piety to Chinese adolescents’ academic autonomous motivation: a mediation model. Front Psychol.

[91] Liu J (2023). Filial piety, love or money? Foundation of old-age support in urban China. J Aging Stud.

[92] Holmes CA, Warelow PJ (1997). Culture needs and nursing: a critical theory approach. J Adv Nurs.

[93] Kaur H, Jutlla K, Moreland N, Read K (2010). How a link nurse ensured equal treatment for people of Asian origin with dementia. Nurs Times.

